# Lesion-directed quantitative EEG after reperfusion therapy is associated with malignant cerebral edema and functional outcomes in acute ischemic stroke: a prospective exploratory cohort study

**DOI:** 10.3389/fneur.2026.1903165

**Published:** 2026-10-30

**Authors:** Yu-Hsuan Wu, Chia-Yen Lin, Jin-An Huang

**Affiliations:** Taichung Veterans General Hospital, Taichung, Taiwan

**Keywords:** cerebral infarction, functional recovery, malignant cerebral edema, qEEG, reperfusion therapy

## Abstract

**Background:**

Ischemic stroke causes electrophysiological slowing within minutes which may precede detectable structural changes, making electroencephalography (EEG) a sensitive bedside marker of tissue at risk. Prior quantitative EEG (qEEG) studies often averaged bilateral or frontal activity, focused on thrombectomy, and were recorded at 24 h. We tested whether a lesion-directed alpha-to-delta ratio (lesion-ADR) within 72 h after reperfusion is associated with malignant cerebral edema (MCE) and functional outcomes.

**Methods:**

In a prospective single-center cohort of anterior-circulation ischemic stroke treated with intravenous alteplase (IV-rtPA), endovascular thrombectomy (EVT), or both, a 7-min EEG was obtained ≤72 h after reperfusion. Lesion-ADR was defined as the mean ADR of ipsilesional electrodes; the reperfusion-to-EEG interval (ΔT) was recorded. The primary outcome was MCE (midline shift ≥5 mm and/or decompressive hemicraniectomy). Secondary outcomes were favorable functional outcomes at discharge and 90 days (modified Rankin Scale [mRS] 0–2). Model discrimination was assessed using ROC analysis and AUC with bootstrap internal validation. Adjusted associations were assessed using parsimonious Firth penalized logistic regression models.

**Results:**

Forty-nine patients were included. For MCE, the combined lesion-ADR + ΔT model achieved an apparent AUC of 0.888 (95% CI 0.765–0.960); bootstrap internal validation yielded an optimism-corrected AUC of 0.889. For discharge independence (mRS ≤ 2; *n* = 9), the ADR-only model showed an apparent AUC of 0.899. Lesion-ADR remained associated with favorable outcome after separate adjustment for admission NIHSS (OR 1.438 per 0.1-unit increase; *p* = 0.009) and reperfusion treatment modality (OR 1.367; *p* = 0.022). For 90-day independence (*n* = 13), the ADR-only AUC was 0.759 (95% CI 0.615–0.869) and the ADR-only model showed moderate discrimination for 90-day functional independence. The association with 90-day outcome was attenuated after accounting for admission NIHSS (OR 1.245 per 0.1-unit increase; *p* = 0.088).

**Conclusion:**

A brief lesion-directed ADR obtained within 72 h after reperfusion was associated with functional outcomes, while its combination with EEG timing showed encouraging discrimination for MCE in this cohort. These exploratory findings suggest lesion-directed qEEG may serve as a potential adjunctive biomarker after reperfusion therapy and warrant validation in larger cohorts.

## Introduction

Ischemic stroke triggers a rapid cascade in which perfusion failure, ionic disequilibrium, and electrical slowing develop in minutes. Because electrophysiological abnormalities may emerge early during cerebral ischemia, scalp electroencephalography (EEG) is highly sensitive to early disturbances in cerebral blood flow and metabolism (1, 2). EEG is non-invasive, inexpensive and provides millisecond temporal resolution; however, conventional visual interpretation requires specialist expertise and is prone to inter-rater variability (3). Therefore, quantitative EEG (qEEG) has been proposed as an objective bedside biomarker of tissue at risk (4). Global or frontal delta-to-alpha metrics, such as the delta-to-alpha ratio (DAR), the delta-theta/alpha-beta ratio (DTABR) and related symmetry indices, have been shown to correlate with 24-h neurological deterioration and 90-day disability. Beyond these ratio-based measures, acute ischemic stroke is characterized by a shift toward slower-frequency activity, with increased delta and theta activity and reductions in faster alpha and beta activity, particularly over the affected hemisphere. These frequency-specific alterations have been associated with cerebral hypoperfusion, neurological deficit, and functional outcome and provide a physiological basis for composite qEEG measures such as DAR, DTABR, and ADR (3–5). Other qEEG approaches, including brain symmetry indices, have also been investigated as markers of post-stroke impairment and recovery (6, 7). Emerging evidence also supports spatially targeted qEEG. In a cohort of patients with middle cerebral artery (MCA) ischemic stroke, relative theta power derived from three ipsilesional posterior electrodes predicted subsequent cognitive impairment with 77% accuracy and outperformed global 19-lead indices (8). Importantly, this finding was related specifically to post-stroke cognitive impairment rather than malignant cerebral edema (MCE), which was not evaluated in that study (8).

Nevertheless, there are still important uncertainties. First, most studies average bilateral activity or restrict the analysis to frontal leads, potentially diluting low-frequency power that is maximal over the infarcted hemisphere (5, 9). Second, the existing cohorts are dominated by patients who underwent endovascular thrombectomy (EVT), with EEG frequently obtained within relatively narrow post-procedural time windows; whether qEEG retains prognostic relevance after intravenous alteplase (IV-rtPA) or across a broader early post-reperfusion interval remains unclear (5, 10). Third, the relationship between lesion-directed qEEG and MCE, a potentially life-threatening complication requiring intensive neurocritical monitoring and, in selected patients, decompressive surgery, remains insufficiently characterized (5).

To address these gaps, we prospectively recorded a 7-min 21-lead EEG (10–20 montage plus T1/T2) within 72 h of reperfusion therapy with IV-rtPA, EVT or combined therapy, and selected lesion-directed alpha-to-delta ratio (lesion-ADR) *a priori* to characterize the balance between alpha activity and pathological delta slowing within the infarcted hemisphere. The present study was not designed to compare the relative prognostic performance of lesion-ADR with other qEEG indices. Lesion-ADR was defined as the mean ADR across all electrodes on the infarcted side. We also measured the interval between reperfusion and EEG (ΔT) to explore the potential time dependency of lesion-ADR. MCE was selected as the primary endpoint because it represents an early, potentially life-threatening complication following anterior-circulation infarction with direct implications for neurocritical monitoring and consideration of decompressive intervention. Favorable functional outcomes at discharge and 90 days were evaluated as secondary endpoints to explore the broader relationship between lesion-ADR and functional recovery.

To our knowledge, this is the first prospective study to evaluate lesion-directed qEEG in the early post-reperfusion period in different treatment modalities and to investigate its associations with MCE and functional recovery. By providing lesion-specific electrophysiological information complementary to conventional clinical assessment, this approach may help characterize early recovery potential and evolving neurological injury after reperfusion therapy, while requiring validation in larger independent cohorts.

## Methods

### Study design and participants

This single-center prospective cohort study was conducted at Taichung Veterans General Hospital (TCVGH) from February 2023 to March 2025. Consecutive adults (≥ 20 years) with acute ischemic stroke of the anterior circulation territory who received reperfusion therapy, IV-rtPA, EVT, or both, were selected for enrollment. According to institutional protocols, IV-rtPA was administered within 4.5 h and EVT within 24 h after the onset of symptoms. Large-vessel occlusion (LVO) was confirmed using CTA and/or CT perfusion imaging. Posterior-circulation strokes were excluded after confirmation of CTA/MRA because the lesion-directed qEEG approach was designed to quantify hemispheric cortical electrophysiological abnormalities; brainstem and cerebellar infarctions may have limited or distinct scalp EEG manifestations and different patterns of edema and neurological deterioration. The study was approved by the Institutional Review Board of TCVGH (CE22262B, CE24295C); written informed consent was obtained from each participant or a legally authorized representative.

### Clinical and imaging data

Baseline variables included age, sex, hypertension, diabetes mellitus, atrial fibrillation (AF), and any prehospital IV-rtPA. Laboratory measurements comprised admission plasma glucose, neutrophil-to-lymphocyte ratios (NLR) at admission and post-reperfusion, D-dimer and low-density lipoprotein cholesterol (LDL-C). These variables were collected to characterize complementary aspects of metabolic stress, systemic inflammatory activation, coagulation activity, and underlying vascular risk. The initial severity of the stroke was assessed using the National Institutes of Health Stroke Scale (NIHSS).

Stroke etiology was classified with the Trial of ORG 10172 in Acute Stroke Treatment (TOAST) criteria (large-artery atherosclerosis, cardioembolism, or other/undetermined). The success of EVT reperfusion was defined as a modified Treatment in Cerebral Infarction (mTICI) score ≥2b. Follow-up NIHSS was recorded 24 h after reperfusion.

Dual-energy CT was obtained 24 h after EVT to identify the hemorrhagic transformation. Additional MRI or CT scans were performed when clinically indicated to assess MCE, defined as a midline shift ≥ 5 mm in an axial image and/or the need for decompressive hemicraniectomy (11). For patients who developed MCE, the time of the first imaging study meeting the predefined MCE criterion was recorded to determine its temporal relationship with EEG acquisition. All images were reviewed by a neuroradiologist blinded to EEG and outcome data.

According to international guidelines, patients receiving IV-rtPA were observed in an ICU or stroke unit for ≥24 h with neurological and blood pressure monitoring every 15 min, then every 30 min, and then hourly. Patients undergoing EVT were admitted to critical care with blood pressure maintained at ≤180/105 mm Hg for the first 24 h (12). There were no missing data for primary predictors or outcomes, and 90-day functional outcome data were available for all participants, with no loss to follow-up.

### Measurement

The patients underwent EEG within 72 h after reperfusion using the international 10–20 system (21 electrodes), supplemented by bilateral anterior temporal electrodes (T1/T2). The signals were recorded in the NicoletOne^™^ EEG System, sampled at 500 Hz, and band-pass filtered from 0.5 to 70 Hz. EEG recordings were performed for at least seven minutes during clinically stable wakeful states without sedative infusion whenever feasible. GCS and detailed sedation status at the time of EEG were not prospectively collected as prespecified study variables and therefore could not be systematically analyzed. The raw traces were reviewed by a board-certified clinical neurophysiologist according to international criteria. Segments containing prominent movement, muscle, electrode, or other nonphysiological artifacts were excluded from quantitative analysis. The proportion of rejected epochs was not prospectively recorded. No predefined numerical artifact-rejection threshold was documented, and the number of excluded epochs could not be reliably reconstructed retrospectively. Offline analysis was performed with Persyst^™^ software. Power in the delta (1–4 Hz) and alpha (8–13 Hz) bands was calculated using a trend of the power ratio based on the fast Fourier transform (FFT) (sampling rate 64 Hz; 128 points per 2-s window; eight windows per epoch; overlapping windows; smoothing factor three; power scale μV/Hz). The ADR was averaged over the seven-minute period. FFT power ratio graphs were generated for each hemisphere. Lesion-ADR was defined as the mean ADR across eight bipolar derivations over the infarcted hemisphere (left: Fp1-F7, F7-T3, T3-T5, T5-O1, Fp1-F3, F3-C3, C3-P3, P3-O1; right: Fp2-F8, F8-T4, T4-T6, T6-O2, Fp2-F4, F4-C4, C4-P4, P4-O2). EEG acquisition was deferred in patients with marked agitation or unstable clinical conditions. EEG acquisition did not require the continuous bedside presence of a neurophysiologist; however, all recordings were subsequently reviewed by a board-certified clinical neurophysiologist for signal quality and artifact identification. Quantitative analysis was performed offline using Persyst^™^ software, allowing automated calculation of ADR after appropriate signal-quality review.

### Statistical analyses

Continuous variables are summarized as median (interquartile range, IQR). Two-group comparisons of continuous data were performed with the independent sample *t*-test (Welch correction when variances were unequal) or the Mann–Whitney U test, as appropriate. Categorical variables were compared with the Chi-square test or Fisher’s exact test when expected cell counts were < 5. Given the limited number of outcome events, adjusted analyses were performed using Firth penalized logistic regression with a parsimonious covariate structure to reduce small-sample bias and the risk of overfitting. Lesion-ADR was evaluated as a continuous variable and scaled per 0.1-unit increase. For functional outcomes, admission NIHSS and reperfusion treatment modality were evaluated separately as clinically relevant potential confounders. Results are reported as odds ratios (ORs) with 95% confidence intervals (CIs).

Discrimination was assessed using receiver operating characteristic (ROC) curves and the area under the curve (AUC) with 95% CIs. Bootstrap resampling with 1,000 iterations was used for internal validation to estimate optimism-corrected AUCs. In addition, bootstrap-derived AUCs and 95% CIs were obtained to assess the uncertainty of the discrimination estimates. For MCE, the discriminatory performance of lesion-ADR alone, the reperfusion-to-EEG interval (ΔT) alone, and their combination was examined. Pairwise differences in AUC were explored using the DeLong test. To address the temporal relationship between EEG acquisition and MCE, a sensitivity analysis was performed after excluding the patient in whom MCE was documented before EEG acquisition. To assess potential confounding by reperfusion treatment modality, an additional sensitivity analysis of the discharge functional outcome was restricted to patients treated with EVT with or without IV-rtPA. The Youden index (sensitivity + specificity – 1) was used to derive exploratory cutoff points (p*) where reported. All tests were two-tailed and *p* ≤ 0.05 was considered statistically significant. The analysis was performed with SPSS version 22.0 (IBM Corp., Armonk, NY, United States); receiver operating characteristic (ROC) curves and additional graphics were produced with the integrated SPSS charting module. Firth penalized logistic regression and bootstrap analyses were performed using R version 4.5.2 with the logistf and pROC packages, respectively. Pairwise AUC comparisons using the DeLong test were performed using MedCalc Statistical Software version 20.0. Model calibration was not formally assessed because the small number of outcome events precluded reliable estimation of calibration.

### Outcomes

The primary outcome was MCE. Secondary outcomes were favorable functional status at discharge and at 90 days, defined as a modified Rankin Scale (mRS) 0–2.

## Results

### Association between lesion-ADR and MCE

A total of 49 patients with anterior-circulation infarctions were analyzed (median age 72 [IQR 62–80] years; 53.1% men). Reperfusion therapy consisted of EVT ± IV-rtPA in 42 patients (85.7%) and IV-rtPA alone in 7 (14.3%).

Table 1 summarizes the baseline characteristics by MCE status. Age, sex, and major vascular risk factors were similar (all *p* > 0.50). The median lesion-ADR was 0.70 (0.50–0.90) in patients without MCE and 0.60 (0.40–0.70) in those who developed MCE (*p* = 0.145). AF and cardioembolic type were more frequent in the MCE group (85.7% vs. 45.2%; *p* = 0.098). The ΔT tended to be shorter in patients with MCE (22.2 h vs. 29.3 h), but the difference did not reach significance (*p* = 0.067) (see Figure 1).

**Table 1:** Baseline characteristics between patients with malignant cerebral edema and without.

Variable	Total (*n* = 49)	No MCE (*n* = 42)	MCE (*n* = 7)	*p* value
Age, years	72.0 (62.0–80.0)	72.0 (61.0–81.0)	71.0 (67.0–78.0)	0.830
Sex				0.692
Female	23 (46.9%)	19 (45.2%)	4 (57.1%)	
Male	26 (53.1%)	23 (54.8%)	3 (42.9%)	
Hypertension	33 (67.3%)	29 (69.0%)	4 (57.1%)	0.668
Diabetes	14 (28.6%)	12 (28.6%)	2 (28.6%)	1.000
Atrial fibrillation	25 (51.0%)	19 (45.2%)	6 (85.7%)	0.098
Admission NIHSS	16.0 (9.5–20.5)	14 (8.75–20)	19 (16.0–21.0)	0.116
D-dimer, μg/mL	1.9 (0.6–5.4)	1.9 (0.6–5.0)	2.3 (1.0–9.0)	0.836
LDL-C, mg/dL	98.0 (85.0–129.0)	98.0 (84.3–127.0)	99.0 (85.0–149.0)	0.710
Glucose at admission, mg/dL	137.0 (110.5–158.0)	132.5 (108.8–150.0)	158.0 (136.0–164.0)	0.113
NLR (baseline)	3.7 (1.8–6.2)	3.6 (1.8–5.8)	6.1 (3.2–8.5)	0.243
NLR (post-reperfusion)	7.6 (4.5–12.6)	7.6 (3.7–11.3)	12.4 (6.6–18.3)	0.097
IV-rtPA alone	7 (14.3%)	7 (16.7%)	0 (0.0%)	0.573
Good recanalization (mTICI ≥2b)	32 (76.2%)	27 (77.1%)	5 (71.4%)	1.000
Lesion-ADR	0.7 (0.5–0.8)	0.7 (0.5–0.9)	0.6 (0.4–0.7)	0.145
Cardioembolic type	25 (51.0%)	19 (45.2%)	6 (85.7%)	0.098
Large-artery atherosclerosis type	14 (28.6%)	13 (31.0%)	1 (14.3%)	0.656
ΔT, h	27.4 (19.2–41.3)	29.3 (20.0–44.2)	22.2 (7.2–28.5)	0.067

Data are n/N (%) or median (IQR). *p* values from Chi-square test or Fisher exact test for categorical variables and Mann–Whitney U test (or Welch t test where appropriate) for continuous variables. Two-tailed α = 0.05. EEG, electroencephalography; ADR, alpha-to-delta ratio; IV-rtPA, intravenous alteplase; MCE, malignant cerebral edema; TOAST, Trial of ORG 10172 in Acute Stroke Treatment; NIHSS, National Institutes of Health Stroke Scale; NLR, neutrophil-to-lymphocyte ratio; LDL, low-density lipoprotein cholesterol; mTICI, modified Treatment in Cerebral Infarction; IQR, interquartile range, ΔT, reperfusion-to-EEG interval.

**Figure 1:**
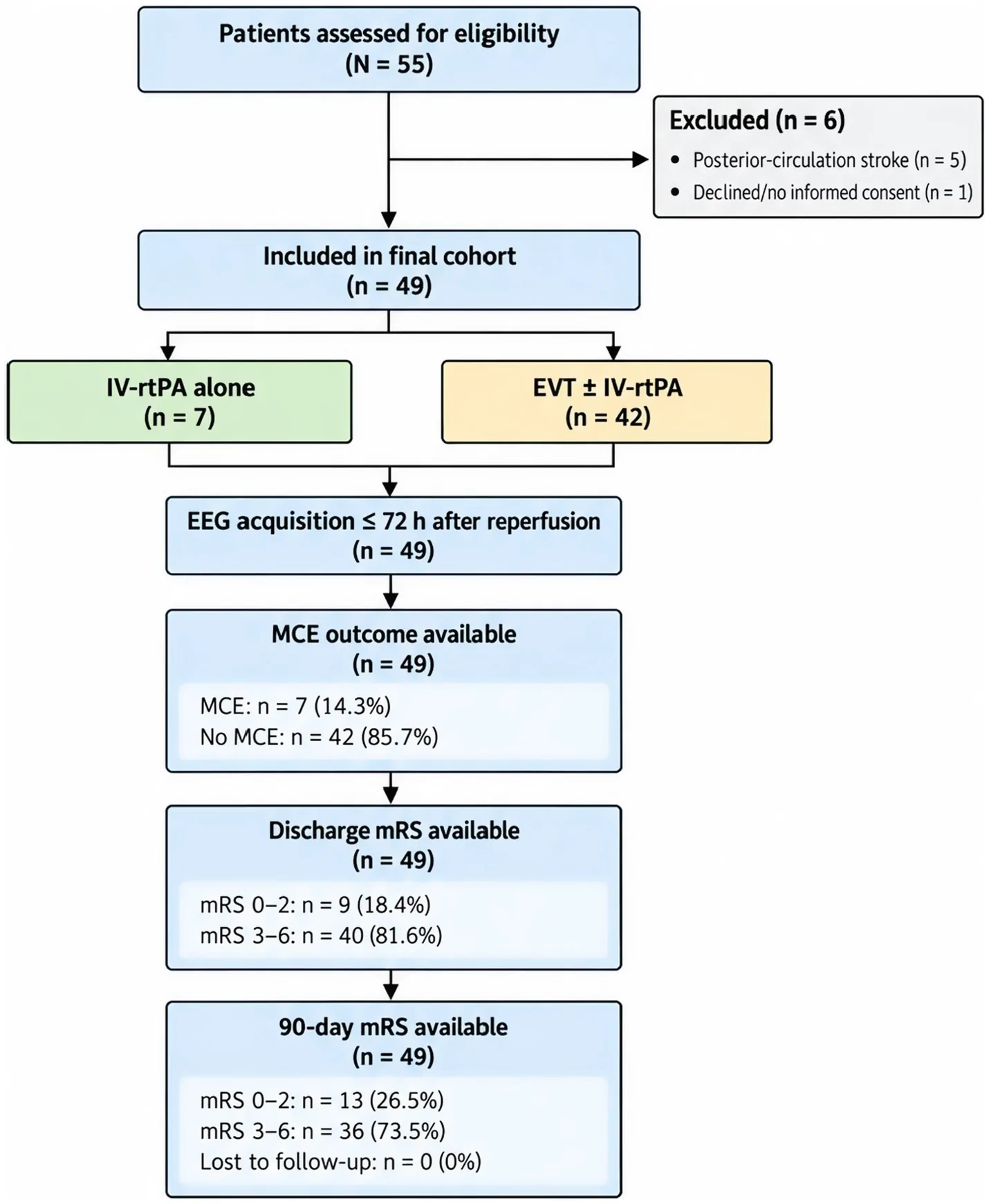
Study flow diagram. EEG, electroencephalography; EVT, endovascular thrombectomy; IV-rtPA, intravenous alteplase; MCE, malignant cerebral edema; mRS, modified Rankin Scale.

Table 2 shows the discriminatory performance of lesion-ADR, the ΔT, and their combination for MCE. Lesion-ADR showed limited discrimination, with an AUC of 0.673 (95% CI 0.525–0.801; *p* = 0.067) whereas ΔT alone produced an AUC of 0.718 (95% CI 0.571–0.837; *p* = 0.019). Combining the two predictors increased the AUC to 0.888 (0.765–0.960; Figure 2), with an optimism-corrected AUC of 0.889 based on 1,000 bootstrap resamples. Bootstrap-derived estimates were similar (AUC 0.889, 95% CI 0.778–0.976). Exploratory pairwise DeLong comparisons showed that the AUC of the combined model was higher than that of lesion-ADR alone (*p* = 0.029) and ΔT alone (*p* = 0.024), whereas the AUCs of lesion-ADR and ΔT alone did not differ (*p* = 0.786). Among the seven patients who developed MCE, EEG acquisition preceded documented MCE in six, whereas MCE was documented before EEG acquisition in one patient. In a sensitivity analysis excluding this patient, the combined lesion-ADR + ΔT model retained high discrimination for subsequent MCE (AUC 0.921, 95% CI 0.805–0.979).

**Table 2:** ROC analysis for malignant cerebral edema.

Predictor/model	AUC	(95% CI)	*p*	p*	Sensitivity	Specificity	Accuracy	PPV	NPV
Lesion-ADR	0.673	(0.525–0.801)	0.067	> 0.13	85.71%	52.38%	57.14%	23.08%	95.65%
ΔT, h	0.718	(0.571–0.837)	0.019	> 0.07	100.00%	42.86%	51.02%	22.58%	100.00%
Lesion-ADR + ΔT	0.888	(0.765–0.960)	< 0.001	> 0.18	85.71%	88.10%	87.76%	54.55%	97.37%

The apparent AUC of the combined lesion-ADR + ΔT model was 0.888, and the optimism-corrected AUC based on 1,000 bootstrap resamples was 0.889. The bootstrap-derived AUC was 0.889 (95% CI 0.778–0.976). EEG, electroencephalography; ADR, alpha-to-delta ratio; AUC, area under the curve; PPV, positive predictive value; NPV, negative predictive value; p*, Youden-optimal model-predicted probability cut-off; ΔT, reperfusion-to-EEG interval.

**Figure 2:**
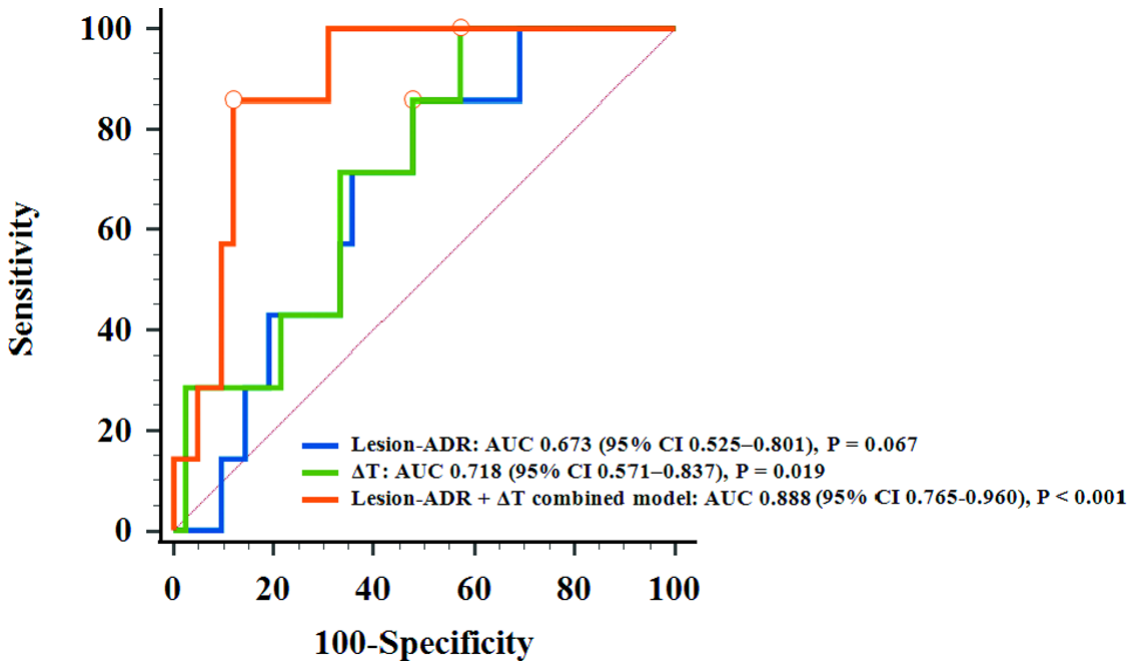
ROC curve of the combined lesion-ADR and ΔT model for predicting malignant cerebral edema. EEG, electroencephalography; ADR, alpha-to-delta ratio; AUC, area under the curve; ROC, receiver operating characteristic; ΔT, reperfusion-to-EEG interval.

### Association between lesion-ADR and favorable discharge outcome

Table 3 compares baseline characteristics between patients who achieved a favorable functional outcome at discharge (mRS ≤ 2; *n* = 9) and those who did not (mRS ≥ 3; *n* = 40). The median age was lower in the favorable outcome group (61 years, IQR 57.5 to 75.5) than in the unfavorable outcome group (73.5 years, 64.8 to 81.0), but the difference did not reach significance (*p* = 0.083). The distribution of sex, hypertension, diabetes, and LDL-cholesterol was similar between cohorts, as was glucose at admission, large-artery atherosclerosis type, the ΔT and the rate of successful angiographic reperfusion (all *p* > 0.10). Several variables differed significantly. AF was present in one single patient with mRS ≤ 2 (11.1%) compared to 24 of 40 patients with mRS ≥ 3 (60.0%; *p* = 0.011). Admission NIHSS was lower in the favorable outcome group (median 8, IQR 4–15) than in the unfavorable outcome group (17, 12.3–20.8; *p* = 0.012). Baseline and post-reperfusion NLR were lower among patients who recovered well (baseline median 1.8 vs. 4.0, *p* = 0.011; post-reperfusion median 2.9 vs. 8.5, *p* = 0.007). IV-rtPA alone was more frequent in the favorable outcome group (55.6% vs. 5.0%; *p* = 0.001). Finally, the lesion-ADR was markedly higher in patients who achieved mRS ≤ 2 (median 1.0, IQR 0.8–1.6) than in those with mRS ≥ 3 (0.6, 0.4–0.7; *p* < 0.001), underscoring its association with early functional recovery.

**Table 3:** Baseline characteristics between patients with favorable functional outcome at discharge and without.

Variable	mRS ≤ 2 (*n* = 9)	mRS ≥ 3 (*n* = 40)	*p* value
Age, years	61.0 (57.5–75.5)	73.5 (64.8–81.0)	0.083
Sex			1.000
Female	4 (44.4%)	19 (47.5%)	
Male	5 (55.6%)	21 (52.5%)	
Hypertension	4 (44.4%)	29 (72.5%)	0.130
Diabetes	3 (33.3%)	11 (27.5%)	0.702
Atrial fibrillation	1 (11.1%)	24 (60.0%)	0.011*
Admission NIHSS	8 (4–15)	17 (12.25–20.75)	0.012*
D-dimer, μg/mL	1.0 (0.6–2.0)	2.4 (0.6–6.1)	0.126
LDL-C, mg/dL	118.0 (95.5–153.0)	93.5 (82.8–123.8)	0.134
Glucose at admission, mg/dL	142.0 (110.5–214.5)	136.5 (109.3–156.8)	0.454
NLR (baseline)	1.8 (0.9–3.1)	4.0 (2.6–6.7)	0.011*
NLR (post-reperfusion)	2.9 (1.6–7.7)	8.5 (4.9–14.0)	0.007**
IV-rtPA alone	5 (55.6%)	2 (5.0%)	0.001**
Good recanalization (mTICI ≥ 2b)	3 (75.0%)	29 (76.3%)	1.000
Lesion-ADR	1.0 (0.8–1.6)	0.6 (0.4–0.7)	<0.001**
Cardioembolic type	2 (22.2%)	23 (57.5%)	0.074
Large-artery atherosclerosis type	2 (22.2%)	12 (30.0%)	1.000
ΔT, h	26.0 (19.1–44.1)	28.9 (19.2–41.8)	0.857
Malignant cerebral edema	0 (0.0%)	7 (17.5%)	0.322

Data are n/N (%) or median (IQR). *p* values from Chi-square test or Fisher exact test for categorical variables and Mann–Whitney U test (or Welch t test where appropriate) for continuous variables. Two-tailed α = 0.05. EEG, electroencephalography; ADR, alpha-to-delta ratio; IV-rtPA, intravenous alteplase; TOAST, Trial of ORG 10172 in Acute Stroke Treatment; NIHSS, National Institutes of Health Stroke Scale; NLR, neutrophil-to-lymphocyte ratio; LDL, low-density lipoprotein cholesterol; mTICI, modified Treatment in Cerebral Infarction; mRS, modified Rankin Scale; IQR, interquartile range; ΔT, reperfusion-to-EEG interval.

Table 4 and Figure 3 depict the ROC curve for discrimination of a favorable functional outcome at discharge. The ADR-only model achieved an AUC of 0.899 (95% CI 0.779–0.966). Bootstrap internal validation with 1,000 resamples yielded an optimism-corrected AUC of 0.899.

**Table 4:** ROC analysis for favorable outcome at discharge.

Predictor/model	AUC	(95% CI)	*p*	p*	Sensitivity	Specificity	Accuracy	PPV	NPV
Lesion-ADR	0.899	(0.779–0.966)	< 0.001	> 0.77	88.89%	82.50%	83.67%	53.33%	97.06%

Bootstrap internal validation with 1,000 resamples yielded an optimism-corrected AUC of 0.899; the bootstrap-derived AUC was 0.899 (95% CI 0.777–0.983). EEG, electroencephalography; ADR, alpha-to-delta ratio; AUC, area under the curve; PPV, positive predictive value; NPV, negative predictive value; p*, Youden-optimal model-predicted probability cut-off.

**Figure 3:**
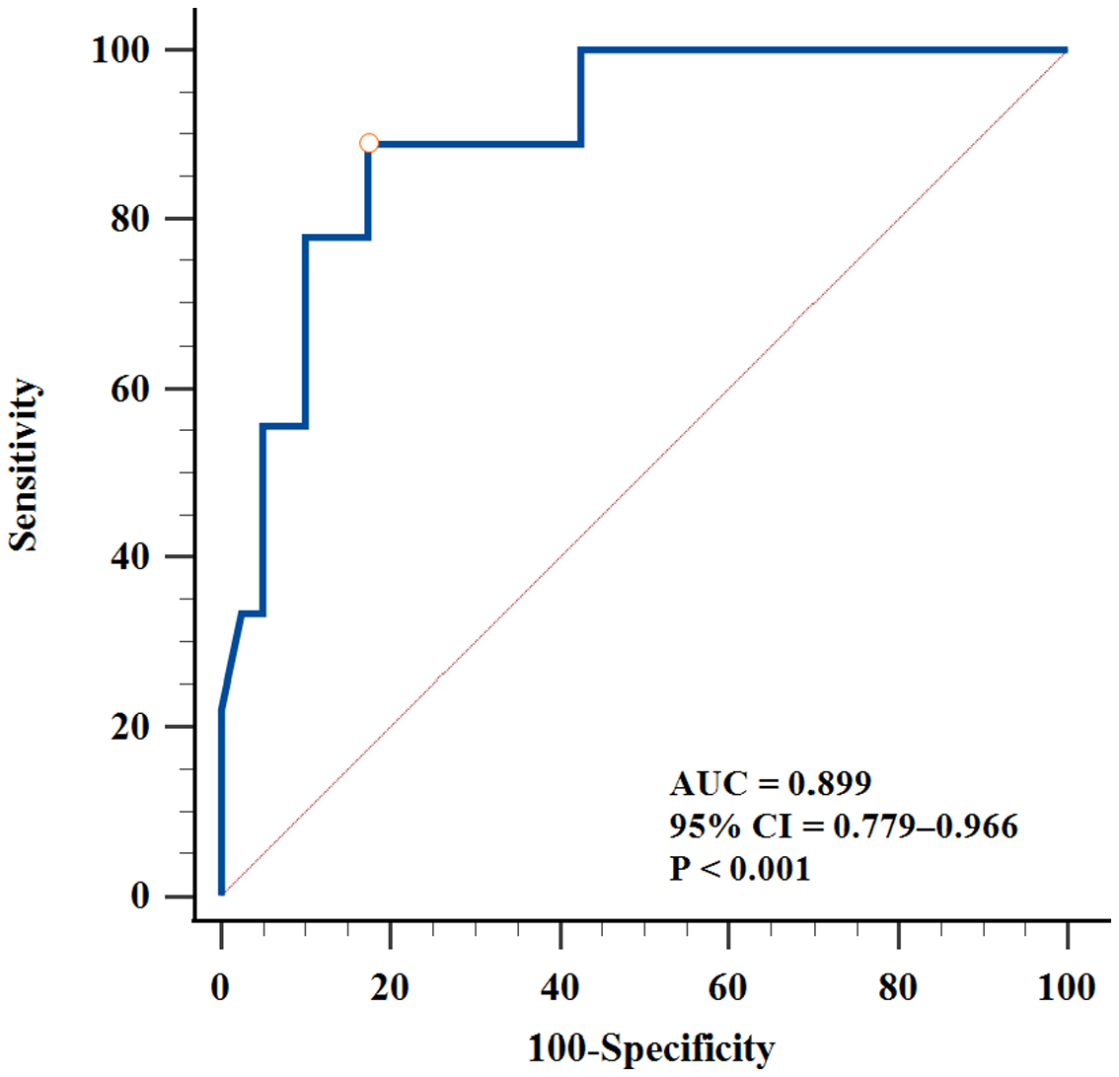
ROC curve for Lesion-ADR (ADR-only model) predicting favorable discharge outcome (mRS ≤ 2). EEG, electroencephalography; ADR, alpha-to-delta ratio; AUC, area under the curve; ROC, receiver operating characteristic.

Table 5 summarizes the Firth penalized logistic regression results. In univariate analyses, AF, admission NIHSS, post-reperfusion NLR, reperfusion treatment modality, and lesion-ADR were associated with favorable discharge outcome (Table 5). Given the limited number of favorable outcome events, adjusted analyses were restricted to parsimonious Firth penalized logistic regression models. Higher lesion-ADR remained associated with favorable discharge outcome after accounting for admission NIHSS (OR 1.438 per 0.1-unit increase, 95% CI 1.087–2.110; *p* = 0.009) and, in a separate model, after accounting for reperfusion treatment modality (OR 1.367 per 0.1-unit increase, 95% CI 1.045–1.932; *p* = 0.022). In a sensitivity analysis restricted to patients treated with EVT with or without IV-rtPA (*n* = 42), lesion-ADR remained higher in patients with favorable than unfavorable discharge outcomes (median 0.856 [IQR 0.7–1.0] vs. 0.564 [IQR 0.4–0.7]; *p* = 0.034), with an AUC of 0.822 (95% CI 0.674–0.923).

**Table 5:** Firth penalized logistic-regression analysis for a favorable functional outcome at discharge.

Variable	Univariate OR (95% CI)	*p* value	Model 1 OR (95% CI)	*p* value	Model 2 OR (95% CI)	*p* value
Age, years	0.938 (0.865–1.006)	0.074				
Sex						
Female	Reference					
Male	1.109 (0.274–4.682)	0.884				
Hypertension	0.319 (0.073–1.320)	0.114				
Diabetes	1.381 (0.288–5.836)	0.669				
Atrial fibrillation	0.119 (0.012–0.604)	0.008*				
Admission NIHSS	0.855 (0.739–0.963)	0.009*	0.976 (0.826–1.137)	0.759		
D-dimer, μg/mL	0.952 (0.760–1.038)	0.326				
LDL-C, mg/dL	1.015 (0.993–1.037)	0.176				
Glucose at admission, mg/dL	1.008 (0.995–1.020)	0.224				
NLR (baseline)	0.708 (0.416–0.990)	0.048*				
NLR (post-reperfusion)	0.795 (0.607–0.957)	0.010*				
IV-rtPA alone	18.822 (3.460–135.545)	<0.001**			5.922 (0.722–50.125)	0.095
Good recanalization (mTICI ≥ 2b)	0.751 (0.106–8.459)	0.788				
Lesion-ADR	1.490 (1.187–2.098)	<0.001**	1.438 (1.087–2.110)	0.009	1.367 (1.045–1.932)	0.022
Cardioembolic TOAST	0.248 (0.042–1.071)	0.062				
Large-artery atherosclerosis	0.760 (0.128–3.376)	0.730				
ΔT, h	0.997 (0.949–1.043)	0.890				

Firth penalized logistic regression. *p < 0.05, **p < 0.01. Lesion-ADR OR is reported per 0.1-unit increase. Model 1 included lesion-ADR and admission NIHSS; Model 2 included lesion-ADR and reperfusion treatment modality. EEG, electroencephalography; ADR, alpha-to-delta ratio; IV-rtPA, intravenous alteplase; TOAST, Trial of ORG 10172 in Acute Stroke Treatment; NIHSS, National Institutes of Health Stroke Scale; NLR, neutrophil-to-lymphocyte ratio; LDL, low-density lipoprotein cholesterol; mTICI, modified Treatment in Cerebral Infarction; mRS, modified Rankin Scale; OR, odds ratio; ΔT, reperfusion-to-EEG interval.

### Association between lesion-ADR and favorable functional 90-day outcome

Table 6 contrasts the baseline characteristics with the functional status. Thirteen out of forty-nine patients (26.5%) achieved mRS 0 to 2. Compared to those with mRS 3–6, the favorable outcome cohort was significantly younger (median 61 [56–74] vs. 74.5 [67.3–81] years, *p* = 0.015) and contained a larger proportion of men (76.9% vs. 44.4%, *p* = 0.044). Fewer had AF (23.1% vs. 61.1%, *p* = 0.019) and their admission NIHSS was markedly lower (median 8 [4–15] vs. 17.5 [13–21], *p* = 0.002). Systemic inflammation was attenuated: baseline NLR was 2.8 (1.0–3.4) versus 4.3 (2.7–7.5) (*p* = 0.005) and post-reperfusion NLR was 3.7 (2.2–6.9) versus 9.1 (5.4–14.9) (*p* < 0.001). IV-rtPA alone was also more common (38.5% vs. 5.6%, *p* = 0.010). The electrophysiological marker differed substantially: the median lesion-ADR was 0.8 (0.7–1.5) in the favorable outcome group and 0.6 (0.4–0.8) in the unfavorable outcome group (*p* = 0.006). Other vascular risk factors, TOAST categories, reperfusion success, MCE, and the ΔT were similar between the groups.

**Table 6:** Baseline characteristics between patients with favorable functional outcome at 90 days and without.

Variable	mRS ≤ 2 (*n* = 13)	mRS ≥ 3 (*n* = 36)	*p* value
Age, years	61.0 (56.0–74.0)	74.5 (67.3–81.0)	0.015*
Sex			0.044*
Female	3 (23.1%)	20 (55.6%)	
Male	10 (76.9%)	16 (44.4%)	
Hypertension	6 (46.2%)	27 (75.0%)	0.086
Diabetes	3 (23.1%)	11 (30.6%)	0.731
Atrial fibrillation	3 (23.1%)	22 (61.1%)	0.019*
Admission NIHSS	8 (4.0–15.0)	17.5 (13.0–21.0)	0.002**
D-dimer, μg/mL	1.2 (0.6–3.7)	2.3 (0.6–11.8)	0.222
LDL-C, mg/dL	102.0 (89.0–139.0)	93.5 (79.8–123.8)	0.253
Glucose at admission, mg/dL	125.0 (108.5–159.0)	137.0 (113–158)	0.541
NLR (baseline)	2.8 (1.0–3.4)	4.3 (2.7–7.5)	0.005**
NLR (post-reperfusion)	3.7 (2.2–6.9)	9.1 (5.4–14.9)	<0.001**
IV-rtPA alone	5 (38.5%)	2 (5.6%)	0.010*
Good recanalization (mTICI ≥2b)	7 (87.5%)	25 (73.5%)	0.655
Lesion-ADR	0.8 (0.7–1.5)	0.6 (0.4–0.8)	0.006**
Cardioembolic type	4 (30.8%)	21 (58.3%)	0.088
Large-artery atherosclerosis type	5 (38.5%)	9 (25.0%)	0.476
ΔT, h	26.0 (19.1–44.8)	28.9 (19.2–40.1)	0.946
Malignant cerebral edema	0 (0.0%)	7 (19.4%)	0.167

Data are n/N (%) or median (IQR). *p* values from Chi-square test or Fisher exact test for categorical variables and Mann–Whitney U test (or Welch t test where appropriate) for continuous variables. Two-tailed α = 0.05. EEG, electroencephalography; ADR, alpha-to-delta ratio; IV-rtPA, intravenous alteplase; TOAST, Trial of ORG 10172 in Acute Stroke Treatment; NIHSS, National Institutes of Health Stroke Scale; NLR, neutrophil-to-lymphocyte ratio; LDL, low-density lipoprotein cholesterol; mTICI, modified Treatment in Cerebral Infarction; mRS, modified Rankin Scale; IQR, interquartile range; ΔT, reperfusion-to-EEG interval.

Figure 4 and Table 7 show the ROC curve for discrimination of 90-day mRS 0–2 (AUC 0.759, 95% CI 0.615–0.869; *p* = 0.003).

**Figure 4:**
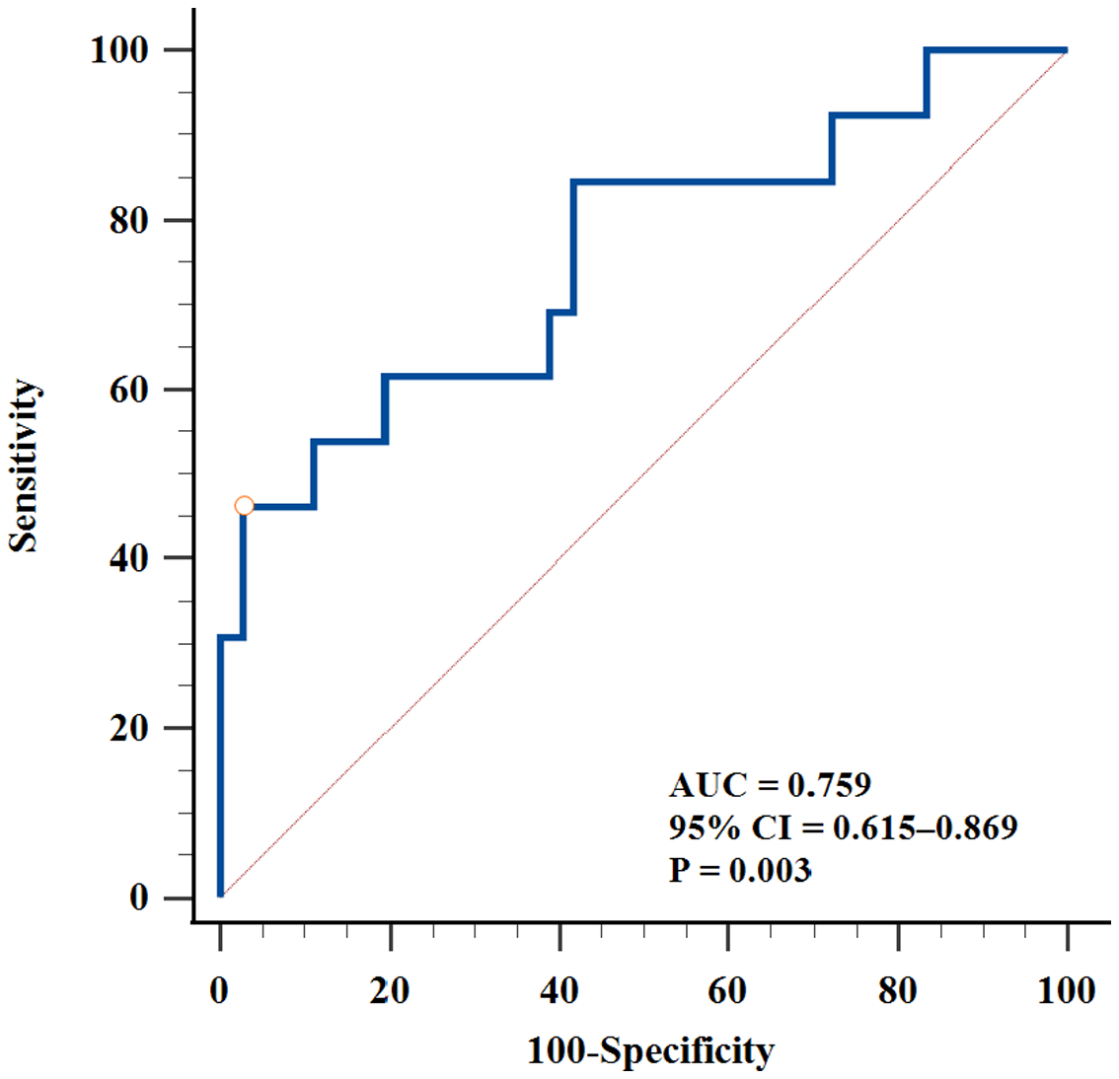
ROC curve for lesion-ADR (ADR-only model) predicting favorable 90-day outcome (mRS ≤ 2). EEG, electroencephalography; ADR, alpha-to-delta ratio; AUC, area under the curve; ROC, receiver operating characteristic.

**Table 7:** ROC analysis for 90-day favorable outcome.

Predictor/model	AUC	(95% CI)	*p*	p*	Sensitivity	Specificity	Accuracy	PPV	NPV
Lesion-ADR	0.759	(0.615–0.869)	0.003	> 0.912	46.15%	97.22%	83.67%	85.71%	83.33%

EEG, electroencephalography; ADR, alpha-to-delta ratio; AUC, area under the curve; PPV, positive predictive value; NPV, negative predictive value; p*, Youden-optimal model-predicted probability cut-off. The optimal cutoff was determined using the Youden index and should be interpreted as exploratory rather than as a clinically validated threshold.

Table 8 details the Firth penalized logistic regression results. Lesion-ADR and several clinical variables were associated with independence in univariate analysis. In a parsimonious Firth penalized logistic regression model accounting for admission NIHSS, the association between higher lesion-ADR and favorable 90-day outcome was attenuated and did not reach statistical significance (OR 1.245 per 0.1-unit increase, 95% CI 0.970–1.720; *p* = 0.088). In a separate model accounting for reperfusion treatment modality, higher lesion-ADR remained associated with favorable 90-day outcome (OR 1.317 per 0.1-unit increase, 95% CI 1.040–1.790; *p* = 0.022).

**Table 8:** Firth penalized logistic regression analysis for a favorable functional outcome at 90 days.

Variable	Univariate OR (95% CI)	*p* value	Multivariable 1 OR (95% CI)		*p* value	Multivariable 2 OR (95% CI)		*p* value
Age, years	0.925 (0.861–0.986)	0.015*						
Sex								
Female	Reference							
Male	3.776 (0.975–16.573)	0.055						
Hypertension	0.324 (0.086–1.156)	0.083						
Diabetes	0.739 (0.184–2.727)	0.651						
Atrial fibrillation	0.225 (0.056–0.809)	0.021*						
Admission NIHSS	0.844 (0.744–0.943)	0.002**	0.904 (0.776–1.031)		0.134			
D-dimer, μg/mL	0.881 (0.701–1.012)	0.104						
LDL-C, mg/dL	1.009 (0.991–1.027)	0.314						
Glucose at admission, mg/dL	1.000 (0.990–1.010)	0.962						
NLR (baseline)	0.670 (0.462–0.924)	0.013*						
NLR (post-reperfusion)	0.738 (0.597–0.881)	<0.001**						
IV-rtPA alone	8.358 (1.618–51.550)	0.011*				2.864 (0.369–22.040)		0.301
Good recanalization (mTICI ≥2b)	2.024 (0.284–23.494)	0.498						
Lesion-ADR	1.331 (1.077–1.733)	0.006**	1.245 (0.970–1.720)		0.088	1.317 (1.040–1.790)		0.022
Cardioembolic type	0.365 (0.097–1.271)	0.116						
Large-artery atherosclerosis type	1.715 (0.470–6.437)	0.414						
ΔT, h	1.001 (0.962–1.040)	0.958						

Firth penalized logistic. *p < 0.05, **p < 0.01. Lesion-ADR OR is reported per 0.1-unit increase. Model 1 included lesion-ADR and admission NIHSS; Model 2 included lesion-ADR and reperfusion treatment modality. EEG, electroencephalography; ADR, alpha-to-delta ratio; IV-rtPA, intravenous alteplase; TOAST, Trial of ORG 10172 in Acute Stroke Treatment; NIHSS, National Institutes of Health Stroke Scale; NLR, neutrophil-to-lymphocyte ratio; LDL, low-density lipoprotein cholesterol; mTICI, modified Treatment in Cerebral Infarction; mRS, modified Rankin Scale; OR, odds ratio; ΔT, reperfusion-to-EEG interval.

## Discussion

In this prospective cohort of anterior-circulation stroke treated with reperfusion therapy, a seven-minute lesion-directed EEG assessment was associated with both MCE and the probability of leaving the hospital with minimal disability. Patients who developed MCE had numerically lower lesion-ADR, and the combined ADR and ΔT model showed encouraging discrimination for MCE in this cohort. These findings support further evaluation of lesion-directed EEG as a potential adjunctive physiological biomarker following reperfusion therapy. In contrast, lesion-ADR showed good discrimination for favorable discharge outcome (AUC 0.899), and its association with favorable outcome remained significant in parsimonious Firth models accounting separately for admission NIHSS and reperfusion treatment modality, supporting its potential role as an adjunctive prognostic biomarker. The persistence of the association between lesion-ADR and discharge outcome after separately accounting for admission NIHSS and reperfusion treatment modality suggests that lesion-directed electrophysiological information may provide complementary physiological information in the early post-reperfusion period. Although ADR was associated with favorable 90-day outcome in univariate analysis, the association was attenuated after accounting for admission NIHSS, suggesting that its relationship with longer-term functional recovery may be less robust than that observed at discharge. Our findings echo a previous study that identified the power of the ipsilateral occipital theta (4.2–7.6 Hz; O1/O2 referenced to Cz) as the only independent predictor of subsequent cognitive impairment, with an optimal criterion ≈ 0.15 (sensitivity 0.80; specificity 0.69), while global 19-lead averages were inferior. Taken together, these findings support the potential value of regionally targeted, reduced-lead EEG approaches for practical bedside deployment (8).

Our findings extend previous work by Shen et al., who reported a frontal DAR AUC of 0.80 for the functional outcome in patients with EVT only examined exactly 24 h after the procedure (5). The present study differs in four methodological aspects that together may partly explain the observed discrimination in this cohort. First, we analyzed a hemispheric ADR derived from all contacts on the infarcted side, including temporal leads, potentially maximizing delta activity over the ischemic hemisphere while avoiding dilution by the contralateral hemisphere. Second, we analyzed IV-rtPA and EVT and treated EEG delay as a continuous variable; the combination of lesion-ADR and ΔT showed higher discrimination for MCE than either measure alone without compromising the bedside feasibility of a seven-minute recording. Third, the outcome was captured at discharge and MCE was strictly defined by ≥ 5 mm midline shift and/or the need for decompressive hemicraniectomy, endpoints that are directly relevant to early neurocritical decisions, but have remained unexplored in previous EEG studies. A fourth distinction is the complete 90-day follow-up, which allowed us to examine the association between lesion-ADR and longer-term functional outcome. Taken together, these methodological refinements yielded encouraging discrimination in this cohort and support further evaluation of lesion-directed EEG approaches in larger studies. The higher proportion of IV-rtPA-alone patients among those with favorable outcomes may partly reflect differences in baseline stroke severity between treatment groups. However, lesion-ADR remained associated with discharge outcome after accounting for reperfusion treatment modality, and an EVT-only sensitivity analysis showed similar directional findings.

Physiologically, a low lesion-ADR reflects a loss of cortical alpha reactivity accompanied by a surge in delta power (13), which may be consistent with neurovascular uncoupling and cytotoxic edema. The observed relationships between ADR, MCE, and functional outcome support the concept that electrical slowing may reflect physiological vulnerability associated with secondary injury (14). The contribution of ΔT to MCE discrimination suggests that the timing of EEG acquisition may influence the prognostic information provided by lesion-ADR. That a single spectral parameter showed consistent associations in this cohort is clinically relevant, because ADR can be calculated automatically and readily incorporated into bedside quantitative EEG workflows.

In our cohort, baseline and post-reperfusion NLR were higher in patients with worse outcomes, with significant differences observed for both discharge and 90-day functional outcomes. This pattern is biologically plausible: after reperfusion, neutrophil-dominated inflammation, endothelial activation, and blood–brain barrier injury intensify, potentially contributing to increased delta activity and edema. Given the limited number of outcome events, NLR was not included in the revised parsimonious multivariable models; therefore, its independent contribution beyond lesion-ADR cannot be determined from the present study. NLR may therefore provide complementary inflammatory context, but its prognostic contribution relative to lesion-ADR, timing, NIHSS, and imaging requires further evaluation in larger cohorts (15–17).

From a clinical perspective, lesion-directed qEEG should be considered an adjunct to, rather than a replacement for, standard neurological assessment and neuroimaging after reperfusion. Because lesion-ADR was measured only once within a 72-h window, the present study cannot determine its temporal evolution or recommend an optimal EEG time point. Future studies should therefore evaluate predefined or serial EEG time points and determine whether lesion-ADR provides additional prognostic information beyond established clinical and imaging predictors before clinical implementation. At present, these findings should be interpreted as exploratory and hypothesis-generating. The number of MCE events was limited (*n* = 7), and although bootstrap internal validation was performed, model estimates may remain unstable and subject to overfitting. The near-identical apparent and optimism-corrected AUCs should not be interpreted as evidence of model robustness, particularly given the small number of outcome events; external validation remains necessary. The wide confidence intervals observed in regression analyses reflect this limitation. Accordingly, the exploratory thresholds identified in this study should not yet be considered definitive clinical decision boundaries and require external validation in larger, multicenter cohorts.

This study has several additional limitations. It was conducted at a single center and included only anterior-circulation strokes, limiting generalizability to posterior-circulation infarctions. EEG acquisition occurred within a flexible 72-h window rather than at a standardized time point, introducing temporal heterogeneity. In addition, GCS and detailed sedation status at the time of EEG were not prospectively collected, and residual confounding related to level of consciousness or sedative exposure cannot be excluded because both may influence alpha and delta activity. In one patient, MCE was documented before EEG acquisition, limiting the temporal interpretation of EEG as an antecedent marker; however, exclusion of this patient in a sensitivity analysis yielded similar findings. Nevertheless, this design reflects real-world clinical practice and enhances the pragmatic applicability of bedside EEG monitoring. External validation in independent cohorts is required before lesion-ADR can be incorporated into routine neurocritical decision-making. Model calibration was not formally assessed and should be evaluated in future validation studies. The regression estimates should be interpreted cautiously given the limited sample size and low event counts. Threshold-based analyses were exploratory and intended primarily for internal model interpretation. In addition, the absence of prospectively recorded quantitative artifact-rejection thresholds and rejected-epoch proportions limits the reproducibility of the qEEG preprocessing procedure.

Despite these limitations, the present findings provide prospective evidence that a short, lesion-directed EEG assessment may offer prognostic information after reperfusion therapy. Because ADR can be automatically computed in a clinically practical timeframe, it may represent a scalable biomarker for future multicenter validation studies. Future studies integrating lesion-directed qEEG with imaging and blood biomarkers may further improve individualized prognostic stratification after reperfusion therapy.

## Data Availability

The raw data supporting the conclusions of this article will be made available by the authors, without undue reservation.

## Glossary

qEEG: quantitative electroencephalography
ADR: alpha-to-delta ratio
EVT: endovascular thrombectomy
IV-rtPA: intravenous alteplase
LVO: large vessel occlusion
MCE: malignant cerebral edema
TOAST: Trial of ORG 10172 in Acute Stroke Treatment
LAA: large artery atherosclerosis
AF: atrial fibrillation
IQR: interquartile range
aOR: adjusted odds ratio
CI: confidence interval
TCVGH: Taichung Veterans General Hospital
NIHSS: National Institutes of Health Stroke Scale
mRS: modified Rankin Scale
NLR: neutrophil-to-lymphocyte ratios
mTICI: modified Treatment in Cerebral Infarction
LDL: low-density lipoprotein cholesterol
AUC: area under the curve
PPV: positive predictive value
NPV: negative predictive value
ROC: receiver operating characteristic
p*: Youden-optimal model-predicted probability cut-off
ΔT: reperfusion-to-EEG interval
MCA: middle cerebral artery
